# Predictive efficacy of PASP combined with NT-proBNP for outcomes in pregnant women with severe cardiovascular disease: a single-centre retrospective observational study

**DOI:** 10.1186/s12884-026-09288-7

**Published:** 2026-05-22

**Authors:** Dan Tian, Dandan Chen, Min Tang, Dawei Lin, Qi Jin, Fan Yang, Yang Zhan, Daxin Zhou, Dehong Kong, Jiarong Zhang, Qianzhou Lv, Lihua Guan, Junbo Ge

**Affiliations:** 1 Department of Pharmacy, Zhongshan Hospital Fudan University, Shanghai, China; 2 Department of Cardiology, Shanghai Institute of Cardiovascular Diseases, Zhongshan Hospital, Fudan University, 180 Fenglin Road, Xuhui District, Shanghai, 200032 China; 3 National Clinical Research Center for Interventional Medicine, 180 Fenglin Road, Xuhui District, Shanghai, 200032 China; 4 Department of Radiology, Zhongshan Hospital, Fudan University, Shanghai, 200032 China; 5 Shanghai Institute of Medical Imaging, Shanghai, 200032 China; 6 Department of Pharmacy, Shanghai Geriatric Medical Center, Shanghai, 201100 China; 7 Department of Echocardiography, Zhongshan Hospital, Fudan University, Shanghai, China; 8 Department of Obstetrics and Gynecology, Zhongshan Hospital, Fudan University, Shanghai, 200032 China

**Keywords:** Congenital heart disease, Pregnancy, Pulmonary hypertension, Pulmonary arterial systolic pressure

## Abstract

**Objective:**

To analyze maternal complications and pregnancy outcomes in pregnant women with severe cardiovascular disease (CVD), and explore the predictive value of pulmonary artery systolic pressure (PASP) and N-terminal pro-B-type natriuretic peptide (NT-proBNP) for these outcomes.

**Method:**

A retrospective observational study was conducted on pregnant women with severe CVD who were admitted to our hospital between December 2019 to August 2025. Demographic data, delivery mode, laboratory and imaging examination results, and post-partum follow-up data were collected. The primary outcome was CVD-related readmission or death during follow-up.

**Results:**

A total of 158 patients (168 pregnancies) were included, with median follow-up of 1.8 (interquartile range 3.0) years. Among them, 86 cases achieved successful delivering, including 14 vaginal deliveries and 72 cesarean sections. Compared with the PASP < 50 mmHg group, the PASP ≥ 50 mmHg group had a significantly higher medical abortion rate (34.5% vs. 12.2%), and a notably lower average birth weight (2247.5 g vs. 3042.5 g, *P* = 0.007). ROC curve analysis revealed that the combined PASP + NT-proBNP index exhibited the highest predictive efficacy for the primary outcome (AUC = 0.748, *P* = 0.001), followed by NT-proBNP alone (AUC = 0.724, *P* = 0.003) and PASP alone (AUC = 0.664, *P* = 0.028). Kaplan-Meier survival analysis demonstrated that pregnant women with elevated PASP (≥ 44.5 mmHg; HR = 3.456, 95% CI: 1.367–8.734; *P* = 0.009) or NT-proBNP (≥ 241.25 pg/mL; HR = 6.863, 95% CI: 2.432–19.370; *P* < 0.001) had a significantly increased risk of cardiovascular-related hospitalization or death during follow-up.

**Conclusion:**

PASP may serve as a reference indicator for guiding perinatal risk assessment in pregnant women with severe CVD, while NT-proBNP could help optimize the long-term prognostic prediction of this population. The combined application of these two parameters might provide a preliminary basis for risk stratification, which may assist in clinical decision-making regarding perinatal management and long-term prognosis monitoring.

**Supplementary Information:**

The online version contains supplementary material available at 10.1186/s12884-026-09288-7.

## What is already known

- Pregnant women with severe CVD are a high-risk group, with well-documented elevated risks of maternal complications, adverse pregnancy outcomes, and long-term cardiovascular events.
- PASP and NT-proBNP have been used for cardiovascular risk stratification in general CVD populations, but their predictive value—especially when combined—for perinatal outcomes and long-term events in this specific pregnant cohort remains unclear.
- Current clinical guidelines lack precise risk stratification tools for these women, often relying on single indicators rather than evidence-based combined metrics.

## What this study adds

- It quantifies outcomes in 158 pregnant women with severe CVD (corresponding to 168 pregnancies), Compared with the PASP < 50 mmHg group, the PASP ≥ 50 mmHg group had a significantly higher medical abortion rate (34.5% vs. 12.2%) and a notably lower average birth weight (2247.5 g vs. 3042.5 g, *P* = 0.007)., thus filling the gap in subtype-specific perinatal data.
- It identifies optimal cut-off values for risk stratification: PASP ≥ 44.5 mmHg (HR = 3.456) and NT-proBNP ≥ 241.25 pg/mL (HR = 6.863) were found to significantly increase the risk of CVD-related hospitalization or death during follow-up. These thresholds provide actionable targets for clinical monitoring and risk stratification.
- It confirms the combined PASP-NT-proBNP index exhibits superior prognostic efficacy (AUC = 0.748) compared with either single marker alone. Based on this, a practical clinical strategy is proposed: using PASP for perinatal risk assessment and NT-proBNP for long-term cardiovascular prognosis prediction, thereby optimizing the clinical management of this high-risk population.

## Introduction

Cardiovascular disease (CVD) is a leading cause of mortality among pregnant women and poses a substantial threat to neonatal health. Severe CVD-related complications can significantly worsen the prognosis for both expectant mothers and their offspring [1–5]. In Western countries, apart from hypertensive disorders of pregnancy (HDP), congenital heart disease (CHD) and valvular heart disease (VHD) are the most prevalent types of CVD during pregnancy, accounting for 57% ~ 66% and 25% ~ 29% of cases, respectively [2, 6]. Untreated CHD increases the risk of pre-pregnancy heart failure (HF), pulmonary hypertension (PH), and cyanosis, which impair maternal and fetal pregnancy outcomes and render fetal risk threefold higher than in women with corrected CHD [7]. Moreover, moderate-to-severe PH complicating CHD and VHD both elevate maternal susceptibility to adverse cardiac events and mortality during pregnancy or early postpartum [2, 8–10].

A data analysis from the European Registry of Pregnancy and Cardiology (ROPAC) spanning 2007 to 2018 shows that the proportion of high-risk pregnancies has increased significantly, rising from 0.7% to 10.9% [2]. In 2019, the Sustainable Development Goals (SDGs) have set a target of reducing the global maternal mortality ratio (MMR) to fewer than 70 deaths per 100,000 live births by 2030 [11]. Currently, China has unified the screening criteria for high-risk pregnant women nationwide, and strengthened the management and referral system for this population, yet lacks precise prognostic tools to support individualized management of pregnant women with severe CVD.

Previous studies have predominantly relied on pregnancy-specific cardiovascular risk scores [e.g., Cardiac Disease in Pregnancy (CARPREG), CARPREG II, Zwangerschap bij Aangeboren HARtAfwijkingen (ZAHARA) scores, and modified WHO (mWHO)] for outcome prediction in women with CHD [12]. However, these scores may have certain limitations, such as static stratification based on disease type, potential overestimation of risk, and poor generalizability across diverse populations [13]. Pulmonary artery systolic pressure (PASP) and N-terminal pro-brain natriuretic peptide (NT-proBNP) are clinically accessible, dynamic biomarkers and common hemodynamic indicators in CVD. Notably, clinical guidelines have explicitly recommended NT-proBNP for preconception and antenatal monitoring in women with CVD [4]. However, targeted data regarding their specific cut-off values and combined predictive value of these two indicators in pregnant women with severe CVD remain insufficient.

Against this background, this study primarily aims to preliminarily explore the prognostic value of combined PASP and NT-proBNP for adverse outcomes (primarily CVD-related readmission or death) in pregnant women with severe CVD, based on clinical data from our center. Specifically, we intend to tentatively determine the optimal cut-off values of these two indicators, verify the predictive efficacy of their combined model, and thereby provide references for clinical perinatal management and long-term prognostic monitoring.

## Method

### Study population

This retrospective observational study aimed to investigate the pregnancy outcomes and postpartum follow-up of women with severe CVD. Severe CVD is defined based on the mWHO 2.0 maternal cardiovascular risk stratification criteria in the 2025 ESC Guidelines for the Management of Cardiovascular Disease and Pregnancy, encompassing mWHO Class III and IV diseases [4]. The medical records of women who were hospitalized in the Department of Obstetrics and Gynecology of Zhongshan Hospital from December 2019 to August 2025 were retrived. Data were extracted from the electronic medical record (EMR) system of pregnant women admitted to the Department of Obstetrics and Gynecology. The inclusion criteria included patients aged ≥ 18 years, admitted to the Department of Obstetrics and Gynecology of our hospital due to pregnancy, and who had a confirmed diagnosis of the following specified CVD: CHD, VHD, PH, HF, aortic dissection, and cardiomyopathy [4]. The exclusion criteria include patients who lack complete in-hospital medical records, those without standardized follow-up, individuals with a history of malignant tumors or other life-threatening diseases, and patients diagnosed with mental illnesses. Patient follow-up was performed through the collection of outpatient medical records and telephone follow-ups, with follow-up censored on August 2025. The primary endpoint was CVD-related hospitalization or death. Death is the most severe adverse outcome in this population, while pregnancy exacerbates cardiac load to trigger acute cardiovascular events and subsequent rehospitalization—a common event significantly linked to long-term mortality risk. Together, these two endpoints cover the full range of adverse outcomes from acute disease exacerbation to death. The study protocol was approved by the Ethics Committee of Zhongshan Hospital, Fudan University (Approval No. B2022-593R).

### Data collection

Patient medical history extracted from the EMR system included the diagnosis time, past medical history, cardiovascular comorbidities, New York Heart Association (NYHA) functional classification, laboratory parameters, and auxiliary examinations (e.g., echocardiography) and main treatment processes. Laboratory indicators, including NT-proBNP and hemoglobin (Hb), were collected from the first measurements performed during the patient’s obstetric hospitalization, which were part of the routine admission screening for high-risk pregnant women. Obstetric data, including gestational age, parity, mode of delivery, anesthesia status, maternal and fetal conditions, were also recorded. PASP is one of the key criteria for diagnosing and grading PH. PASP values obtained via echocardiography or right heart catheterization (RHC) can assist in assessing disease severity [14]. For this study, PASP data were extracted from the echocardiographic examination closest to the patient’s obstetric hospitalization, with the corresponding measurement gestational age consistent with the hospitalization gestational age to reflect the most relevant hemodynamic status during the perinatal period. PASP was estimated using the modified Bernoulli equation in the absence of gradients across the pulmonary valve or right ventricular outflow tract (RVOT) by the following formula: PASP = 4 (TRV)^2^ + RAP. The peak tricuspid regurgitation jet velocity (TRV) was measured via continuous-wave Doppler. Right atrial pressure (RAP) was inferred from the diameter and collapsibility of the inferior vena cava based on the the American Society of Echocardiography (ASE) guidelines [15, 16]. Mild PH is generally defined as a PASP ranging from 30 to 49 mmHg, while moderate-to-severe PH is characterized by a PASP of ≥ 50 mmHg, which typically results in a significant increase in right heart workload. Pregnancy may be manageable in women with well-controlled PH or mild PH (PASP < 50 mmHg) [17]. Data were collected by trained researchers and entered into a preformatted database. All data were double-entered, followed by routine logical validation and consistency checks.

### Statistical analysis

The normality of continuous variables was evaluated using the Shapiro-Wilk test. Continuous variables were summarized as mean±standard deviation (SD) or median (interquartile range) and compared using the independent samples t-test or Mann-Whitney U test, as appropriate. Categorical variables were presented as frequency (incidence) and were analyzed using chi-square test or Fisher’s exact test. Missing data were imputed via multiple imputation with 10 iterations and chained equations. Pregnant women were grouped by based on successful delivery status and the occurrence of outcome events during follow-up to screen for outcome-related risk factors. Variables that were considered clinically important based on existing evidence and clinical practice, together with variables showing *P* < 0.1 in univariate logistic regression and no significant multicollinearity, were entered into the multivariate logistic regression model. Backward stepwise elimination was used for variable selection. Model goodness-of-fit was evaluated by the Hosmer-Lemeshow test. Potential multicollinearity among covariates was assessed using the variance inflation factor (VIF) and tolerance, with VIF > 10 or tolerance < 0.1 indicating significant collinearity. The screened variables were then stratified using optimal cut-off values determined by receiver operating characteristic (ROC) curve analysis. The optimal thresholds were selected based on the Youden index to maximize the predictive efficiency for the composite endpoint. Kaplan-Meier curves were plotted by follow-up duration (years), and cumulative survival differences between groups were assessed via the log-rank test. All statistical analyses were performed using SPSS 26.0 software (2019, IBM Corp., Armonk, NY, USA), and graphs were generated using GraphPad Prism 8 and R 4.4.3 (2025, R Core Team/R Foundation for Statistical Computing, Vienna, Austria). The significance level was set at α = 0.05 (two-tailed test), and a P-value < 0.05 was considered statistically significant.

## Results

### Patient characteristics according to the PASP level

**Fig. 1:**
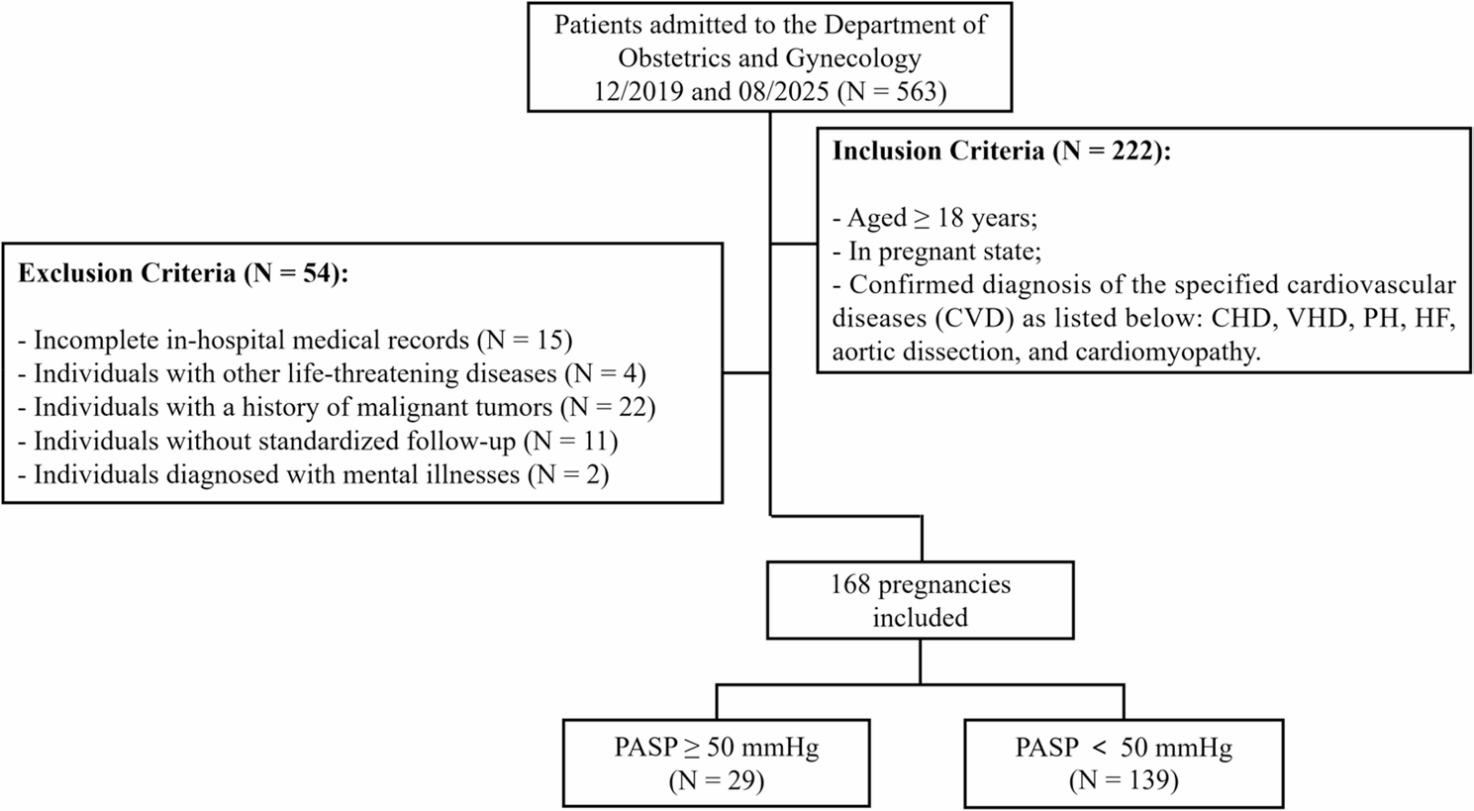
Flowchart of included and excluded patients. CVD, cardiovascular disease; PASP, pulmonary arterial systolic pressure; CHD, congenital heart disease; VHD, valvular heart disease; PH, pulmonary hypertension; HF, heart failure

A total of 158 patients (corresponding to 168 pregnancies) with severe CVD were included (Fig. 1). Among them, 98 (58.3%) had CHD, 72 (42.8%) had VHD, 42 (25.0%) had PH. 5 (3.0%) had aortic dissection. 28 (16.7%) had both CHD and VHD. Other diagnoses included arrhythmia in 11 patients, hypertrophic cardiomyopathy (HCM) / dilated cardiomyopathy (DCM) in 8 patients, and heart failure in 5 patients, etc. 86 patients successfully gave birth, including 14 cases of natural delivery and 72 cases of cesarean section (Table 1 & Supplement Table 1). Patients were classified based on the admission PASP of transthoracic echocardiography. There was no difference in the demographic characteristics (age, reproductive-related conditions) between the two groups (*P* > 0.05). Patients with PASP ≥ 50 mmHg had a significantly higher proportion of diagnosis made during pregnancy than those with PASP < 50 mmHg (44.8% vs. 24.5%, *P* = 0.026). They also showed lower VHD prevalence, higher HF incidence, more severe cardiac dysfunction (37.9% at NYHA class Ⅲ-Ⅳ), higher Hb and NT-proBNP levels, and lower high-sensitivity C-reactive protein (hs-CRP) levels. Additionally, the PASP ≥ 50 mmHg group had significantly higher utilization of loop diuretics, spironolactone, and PAH-targeted drugs. No significant differences existed between the two groups in other medication use, left ventricular ejection fraction (LVEF), and D-dimer.

**Table 1:** Baseline characteristics, management, and outcomes of pregnant women with severe CVD

Variables	Total *N* = 168	PASP ≥ 50 mmHg *N* = 29	PASP < 50 mmHg *N* = 139	*P* value
Demographic Information				
Age, years	31.0 (6.0)	29.0 (7.0)	31.0 (5.0)	0.181
Nulliparous, *n* (%)	61 (36.3)	13 (44.8)	48 (34.5)	0.294
History of Abortion, *n* (%)				
0	95 (56.5)	15 (51.7)	80 (57.6)	0.715
1	44 (26.2)	10 (34.5)	34 (24.5)	
2	18 (10.7)	3 (10.3)	15 (10.8)	
3	5 (3.0)	0 (0.0)	5 (3.6)	
4	6 (3.6)	1 (3.4)	5 (3.6)	
Child, *n* (%)				
0	110 (65.5)	18 (62.1)	92 (66.2)	0.627
1	44 (26.2)	7 (24.1)	37 (26.6)	
2	11 (6.5)	3 (10.3)	8 (5.8)	
3	2 (1.2)	1 (3.4)	1 (0.7)	
4	1 (0.6)	0 (0.0)	1 (0.7)	
Diagnosis made				
Before pregnancy, *n* (%)	121 (72.0)	16 (55.2)	105 (75.5)	0.026
During pregnancy, *n* (%)	47 (28.0)	13 (44.8)	34 (24.5)	
Diagnosis				
CHD, *n* (%)	98 (58.3)	21 (72.4)	77 (55.4)	0.091
No surgery, *n* (%)	51 (52.0)	13 (61.9)	38 (49.4)	0.307
Surgical intervention, *n* (%)	47 (48.0)	8 (38.1)	39 (50.6)	
VHD, *n* (%)	72 (42.9)	7 (24.1)	65 (46.8)	0.025
No surgery, *n* (%)	30 (41.7)	4 (57.1)	26 (40.0)	0.440
Surgical intervention, *n* (%)	42 (58.3)	3 (42.9)	39 (60.0)	
Aortic dissection, *n* (%)	5 (3.0)	0 (0.0)	5 (3.6)	0.589
Arrhythmia, *n* (%)	11 (6.5)	1 (3.4)	10 (7.2)	0.692
HCM/DCM, *n* (%)	8 (4.8)	0 (0.0)	8 (5.8)	0.353
Hypertension, *n* (%)	7 (4.2)	0 (0.0)	7 (5.0)	0.606
HF, *n* (%)	5 (3.0)	3 (10.3)	2 (1.4)	0.037
CAD, *n* (%)	3 (1.8)	0 (0.0)	3 (2.2)	1.000
Endocarditis or myocarditis, *n* (%)	3 (1.8)	0 (0.0)	3 (2.2)	1.000
NYHA class and echocardiography				
I, *n* (%)	90 (53.6)	3 (10.3)	87 (62.6)	0.000
II, *n* (%)	62 (36.9)	15 (51.7)	47 (33.8)	
III, *n* (%)	15 (8.9)	10 (34.5)	5 (3.6)	
IV, *n* (%)	1 (0.6)	1 (3.4)	0 (0.0)	
PASP, mmHg	34.0 (14.0)	79.0 (28.0)	32.0 (8.0)	0.000
LVEF, %	65.0 (5.0)	65.0 (6.0)	65.0 (5.0)	0.281
Laboratory examination				
Hb, g/L	111.0 (21.0)	115.5 (27.0)	111.0 (22.0)	0.028
NT-proBNP, pg/ml	131.5 (188.3)	258.8 (678.4)	124.0 (163.8)	0.002
D-dimer, mg/L	1.4 (2.6)	1.6 (2.3)	1.4 (2.7)	0.272
hs-CRP, mg/L	36.2 (46.6)	16.1 (22.9)	40.4 (46.9)	0.002
UA, µmol/L	263.5 (96.0)	274.0 (71.5)	263.5 (102.0)	0.473
Creatinine, µmol/L	50.0 (10.0)	49.5 (9.5)	50.0 (10.2)	0.105
Medications after discharge				
Anticoagulants	82 (48.8)	11 (37.9)	71 (51.1)	0.198
Antihypertensive drugs	10 (6.0)	1 (3.4)	9 (6.5)	1.000
Loop diuretics	14 (8.3)	7 (24.1)	7 (5.0)	0.001
Spironolactone	7 (4.2)	5 (17.2)	2 (1.4)	0.002
Warfarin	9 (5.4)	2 (6.9)	7 (5.0)	0.654
Iron supplement	31 (18.5)	3 (10.3)	28 (20.1)	0.296
PAH target drugs				
Monotherapy	2 (1.2)	2 (6.9)	0 (0.0)	0.000
Dual-agent Therapy	9 (5.4)	8 (27.6)	1 (0.7)	
Triple-agent Therapy	3 (1.8)	3 (10.3)	0 (0.0)	

Values are presented as median (interquartile range) or *n* (%)

*CVD* cardiovascular disease, *PASP* pulmonary arterial systolic pressure, *CHD* congenital heart disease, *VHD* valvular heart disease, *PH* pulmonary hypertension, *HCM* hypertrophic cardiomyopathy, *DCM* dilated cardiomyopathy, *HF* heart failure, *CAD* coronary heart disease, *NYHA* New York Heart Association, *Hb* hemoglobin, *LVEF* left ventricular ejection fraction, *NT-proBNP* N-terminal-pro Brain natriuretic peptide, *hs-CRP* high-sensitivity C-reactive protein, *UA* uric acid, *PAH* pulmonary arterial hypertension

### Pregnancy outcomes and follow-up results stratified by PASP

**Fig. 2:**
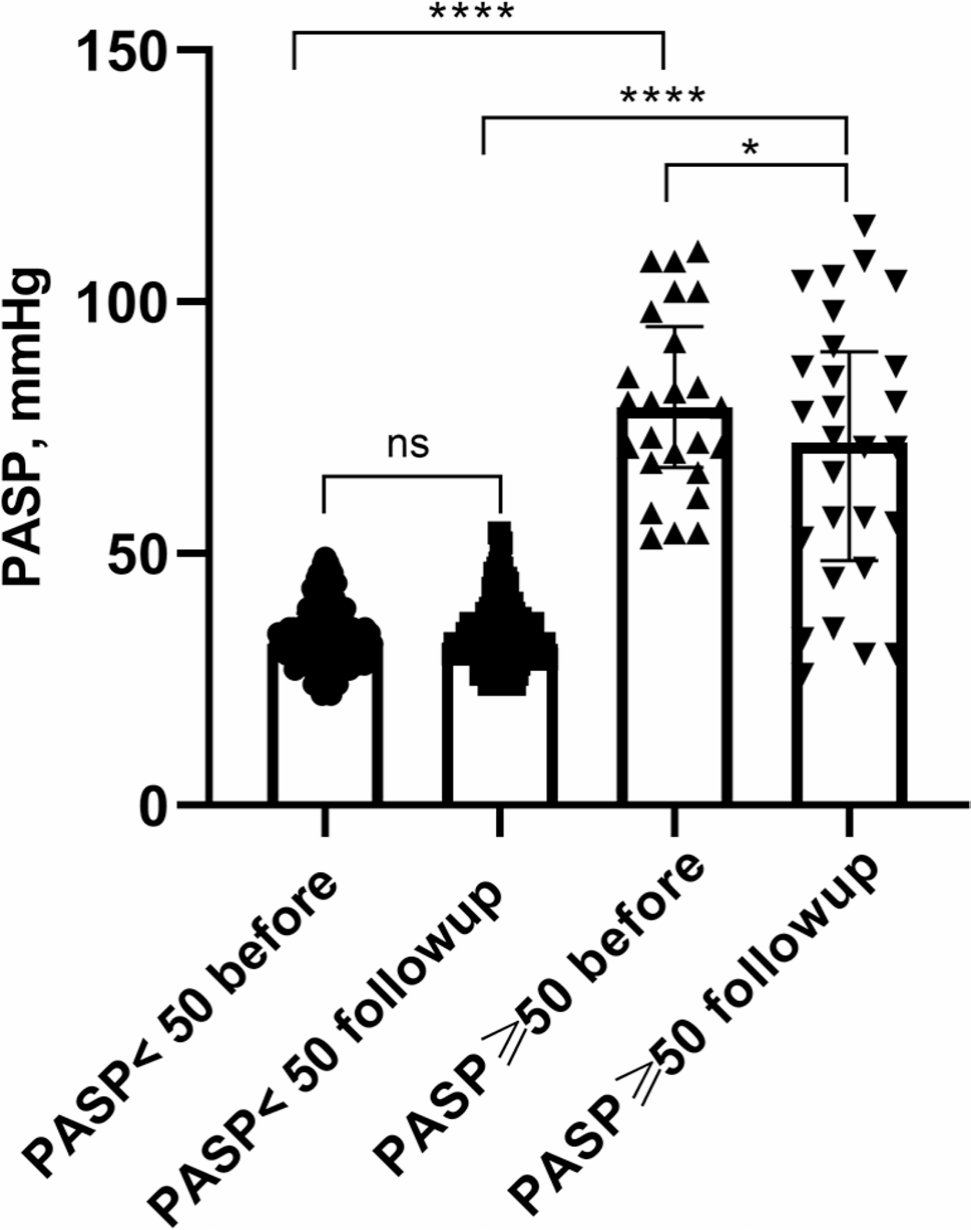
Changes in PASP before and after follow-up in patients with different baseline PASP levels. PASP, pulmonary arterial systolic pressure; ns, not significant; *P < 0.05, ****P < 0.0001

Our data showed that a total of 18 patients experienced CVD-related hospitalization or death events during the follow-up period, of which 9 cases (5.4%) were fatalities (Supplementary Tables 2–3). Intergroup comparisons revealed a statistically significant difference in the distribution of gestational age (*P* = 0.046). Specifically, pregnancies with a PASP of ≥ 50 mmHg and a gestational age of ≤ 20 weeks accounted for the highest proportion (51.7%), whereas those with a gestational age of > 35 weeks constituted a relatively low proportion (31.0%). There was a statistically significant difference in the overall pregnancy outcomes between the two groups (*P* = 0.031), which were mainly reflected in the difference in clinical decision-making related to abortion: the rate of medical abortion was significantly higher in the PASP ≥ 50 mmHg group (34.5%) than in the PASP < 50 mmHg group (12.2%), while the rate of surgical abortion was lower in the PASP ≥ 50 mmHg group. In terms of delivery mode distribution, there was no significant difference between the two groups, specifically, the cesarean section rate was slightly higher in the PASP < 50 mmHg group (44.6%, 62/139) than in the PASP ≥ 50 mmHg group (34.5%, 10/29). Among the 72 cesarean deliveries, the indications included maternal cardiac factors (*n* = 51, 70.8%), obstetric factors (*n* = 13, 18.1%; e.g., cephalopelvic disproportion, preterm labor complicated with fetal distress), and fetal factors (*n* = 8, 11.1%; e.g., non-reassuring fetal heart rate patterns). The birthweight of neonates in the PASP ≥ 50 mmHg group [2247.5 (1569.0) g] was significantly lower than that in PASP < 50 mmHg group [3042.5 (600.0) g] (*P* = 0.007). However, there were no statistically differences in the rates of embryonic arrest/developmental arrest (13.8% vs. 14.4%) or the distribution of anesthesia methods between the two groups (*P* = 0.280). The median follow-up time was 1.8 (IQR 3.0) years. The incidence of composite cardiovascular hospitalization and death events in the PASP ≥ 50 mmHg group (24.1%, 7/29) was significantly higher than in the PASP < 50 mmHg group (7.9%, 11/139) (*P* = 0.010). Results of follow-up echocardiography showed that the PASP level in the PASP ≥ 50 mmHg group was significantly higher than that in the latter group (72.0 mmHg vs. 32.0 mmHg, *P* < 0.001), while there was no statistically significant difference in the LVEF between the two groups (*P* = 0.789) (Table 2; Fig. 2). Supplementary Table 4 presents the changes in PASP between the follow-up period and the hospitalization period in patients with different clinical diagnoses and characteristics. Among these patients, those with PH had a significant decrease in PASP, from 63.5 mmHg to 45.0 mmHg (*P* = 0.0054).

**Table 2:** Pregnancy outcomes and follow-up results of pregnant women with severe CVD stratified by PASP

Variables	Total *N* = 168	PASP ≥ 50 mmHg *N* = 29	PASP < 50 mmHg *N* = 139	*P* value
Gestational age	23.5 (30.0)	15.6 (27.3)	33.0 (30.4)	0.249
Gestational week				
Q ≤ 20 weeks, *n* (%)	80 (47.6)	15 (51.7)	65 (46.8)	0.046
20 < Q ≤ 30 weeks, *n* (%)	5 (3.0)	3 (10.3)	2 (1.4)	
30 < Q ≤ 35 weeks, *n* (%)	9 (5.4)	2 (6.9)	7 (5.0)	
Q > 35 weeks, *n* (%)	74 (44.0)	9 (31.0)	65 (46.8)	
Pregnancy outcome				
Surgical abortion, *n* (%)	55 (32.7)	7 (24.1)	48 (34.5)	0.031
Drug abortion, *n* (%)	27 (16.1)	10 (34.5)	17 (12.2)	
Vaginal delivery, *n* (%)	14 (8.3)	2 (6.9)	12 (8.6)	
Cesarean section, *n* (%)	72 (42.9)	10 (34.5)	62 (44.6)	
Anesthesia				
No, *n* (%)	39 (23.2)	10 (34.5)	29 (20.9)	0.280
Epidural anesthesia, *n* (%)	71 (42.3)	10 (34.5)	61 (43.9)	
General anesthesia, *n* (%)	58 (34.5)	9 (31.0)	49 (35.2)	
Arrested or Suspended embryo growth				
Yes, *n* (%)	24 (14.3)	4 (13.8)	20 (14.4)	1.000
No, *n* (%)	144 (85.7)	25 (86.2)	119 (85.6)	
Birthweight, kg	2972.5 (728.0)	2247.5 (1569.0)	3042.5 (600.0)	0.007
Follow-up duration, years	1.8 (3.0)	1.5 (4.0)	2.0 (2.7)	0.837
Cardiovascular hospitalizations and death events, *n* (%)	18 (10.7)	7 (24.1)	11 (7.9)	0.010
Time of occurrence of endpoint event, years	0.2 (1.0)	0.1 (0.4)	0.3(1.5)	0.892
Follow-up echocardiography				
PASP, mmHg	34.0 (9.0)	72.0 (42.0)	32.0 (6.0)	0.000
LVEF, %	65.0 (5.0)	65.0 (5.0)	65.0 (5.0)	0.789

Values are presented as median (interquartile range) or *n* (%)

*CVD* cardiovascular disease, *PASP* pulmonary arterial systolic pressure, *LVEF* left ventricular ejection fraction

Among the 72 cesarean deliveries, the indications included maternal cardiac factors (*n* = 51, 70.8%; e.g., cardiac insufficiency, severe pulmonary hypertension), obstetric factors (*n* = 13, 18.1%; e.g., cephalopelvic disproportion, preterm labor complicated with fetal distress), and fetal factors (*n* = 8, 11.1%; e.g., non-reassuring fetal heart rate patterns)

### Logistic regression analysis of the influencing factors of successful delivery and follow-up outcomes

Univariate logistic regression analysis identified maternal age, history of abortion, VHD, PASP, and Hb ≥ 11.1 g/dL, were found to exert negative effects on delivery success. By contrast, factors such as CHD, NYHA cardiac function classification, and LVEF showed no statistically significant correlation with successful delivery in pregnant women (*P* > 0.05) (Table 3). A subsequent multivariate logistic regression model was constructed incorporating the following four covariates: history of abortion, VHD, PASP, and Hb levels ≥ 11.1 g/dL. The analysis demonstrated that history of abortion (OR = 0.242, 95% CI: 0.113–0.521, *P* = 0.000), VHD (OR = 0.342, 95% CI: 0.162–0.719, *P* = 0.005), PASP (OR = 0.978, 95% CI: 0.960–0.996, *P* = 0.016), and Hb levels ≥ 11.1 g/dL (OR = 0.293, 95% CI: 0.141–0.611, *P* = 0.001) were independent negative predictors of successful delivery. Table 4 presents the results of logistic regression analysis evaluating factors associated with adverse follow-up outcomes in pregnant women with severe cardiovascular complications. In the univariate analysis, aortic dissection and elevated hs-CRP, and NT-proBNP levels ≥ 200.0 pg/mL were all significantly associated with an increased risk of adverse pregnancy outcomes (all *P* < 0.05). By contrast, elevated PASP was associated with a trend toward increased risk, albeit without statistical significance (OR = 1.020, 95% CI: 1.000–1.041, *P* = 0.054). For the multivariable analysis of adverse follow-up outcomes, a total of four covariates were included: aortic dissection, PASP, NT-proBNP ≥ 200.0 pg/mL, and hs-CRP. Multivariate analysis demonstrated that only three factors were independent risk factors for adverse outcomes: aortic dissection (OR = 61.181, 95% CI: 5.174–723.479, *P* = 0.001), elevated PASP (OR = 1.048, 95% CI: 1.012–1.084, *P* = 0.008), and NT-proBNP levels ≥ 200.0 pg/mL (OR = 5.700, 95% CI: 1.019–31.873, *P* = 0.048).

**Table 3:** Logistic regression analysis of successful delivery in pregnant women with severe cardiovascular complications

Variables	Univariate logistic regression		Multivariate logistic regression	
	OR (95% CI)	*P* value	OR (95% CI)	*P* value
Demographic Information				
Age	0.935 (0.875–0.988)	0.044		
History of Abortion	0.323 (0.171–0.609)	0.000	0.242 (0.113–0.521)	0.000
Diagnosis				
CHD	1.609 (0.868–2.985)	0.131		
VHD	0.417 (0.223–0.780)	0.006	0.342 (0.162–0.719)	0.005
CHD/VHD Surgical correction	0.653 (0.355–1.201)	0.171		
Aortic dissection	1.446 (0.235–8.882)	0.691		
PH	0.938 (0.467–1.887)	0.859		
HCM/DCM	0.951 (0.230–3.936)	0.945		
Hypertension	2.469 (0.465–13.099)	0.288		
HF	1.446 (0.235–8.882)	0.691		
CAD	1.929 (0.172–21.684)	0.595		
Myocarditis and endocarditis	0.471 (0.042–5.291)	0.542		
Echocardiography and laboratory examination				
PASP, mmHg	0.982 (0.966–0.998)	0.030	0.978 (0.960–0.996)	0.016
LVEF, %	0.957 (0.895–1.024)	0.206		
Hb ≥ 11.1 g/dL	0.330 (0.176–0.632)	0.001	0.293 (0.141–0.611)	0.001
NT-proBNP ≥ 200.0pg/ml	0.632 (0.327–1.223)	0.173		
NYHA III/IV class	0.719 (0.255–2.029)	0.533		
Arrested or Suspended embryo growth	0.639 (0.266–1.533)	0.316		

*OR* odds ratio, *CI* confidence interval, *CHD* congenital heart disease, *VHD* valvular heart disease, *PH* pulmonary hypertension, *HCM* hypertrophic cardiomyopathy, *DCM* dilated cardiomyopathy, *HF* heart failure, *CAD* coronary heart disease, *PASP* pulmonary arterial systolic pressure, *LVEF* left ventricular ejection fraction, *Hb* hemoglobin, *NT-proBNP* N-terminal-pro Brain natriuretic peptide, *NYHA* New York Heart Association

**Table 4:** Logistic regression analysis of follow-up outcomes for pregnant women with severe cardiovascular complications

Variables	Univariate logistic regression		Multivariate logistic regression	
	OR (95% CI)	*P* value	OR (95% CI)	*P* value
Demographic Information				
Age	0.950 (0.854–1.057)	0.343		
Nulliparous	0.884 (0.324–2.414)	0.810		
History of Abortion	1.046 (0.391–2.799)	0.928		
History of full-term delivery	0.747 (0.252–2.210)	0.598		
Diagnosis made during pregnancy	2.277 (0.839–6.181)	0.106		
Diagnosis				
CHD	1.138 (0.418–3.098)	0.800		
VHD	1.381 (0.519–3.676)	0.518		
CHD/VHD Surgical correction	0.527 (0.188–1.479)	0.224		
Aortic dissection	14.8 (2.289–95.677)	0.005	61.181 (5.174-723.479)	0.001
PH	1.583 (0.555–4.521)	0.391		
Hypertension	1.412 (0.160-12.437)	0.756		
HF	2.147 (0.227–20.333)	0.505		
CAD	0.000 (0.000–0.000)	0.999		
Myocarditis and endocarditis	0.000 (0.000–0.000)	0.999		
Echocardiography and laboratory examination				
PASP, mmHg	1.020 (1.000-1.041)	0.054	1.048 (1.012–1.084)	0.008
LVEF, %	0.961 (0.883–1.047)	0.364		
Hb ≥ 11.1 g/dL	1.973 (0.702–5.540)	0.197		
NT-proBNP ≥ 200.0 pg/ml	6.005 (2.013–17.915)	0.001	5.700 (1.019–31.873)	0.048
hs-CRP, mg/L	0.966 (0.936–0.997)	0.030		
D-dimer, mg/L	0.930 (0.720–1.200)	0.574		
UA, µmol/L	1.002 (0.998–1.007)	0.278		
Creatinine, µmol/L	0.998 (0.951–1.048)	0.944		
Arrested or Suspended embryo growth	1.229 (0.327–4.611)	0.760		
Successful delivery	0.438 (0.156–1.227)	0.116		

*OR* odds ratio, *CI* confidence interval, *CHD*, congenital heart disease, *VHD* valvular heart disease, *PH* pulmonary hypertension, *HCM* hypertrophic cardiomyopathy, *DCM* dilated cardiomyopathy, *HF* heart failure, *CAD* coronary heart disease, *PASP* pulmonary arterial systolic pressure, *LVEF* left ventricular ejection fraction, *Hb* hemoglobin, *NT-proBNP* N-terminal-pro Brain natriuretic peptide, *hs-CRP* high-sensitivity C-reactive protein, *UA* uric acid, *NYHA* New York Heart Association

### PASP serves as a predictive factor for the follow-up outcomes of pregnant women with severe CVD

**Fig. 3:**
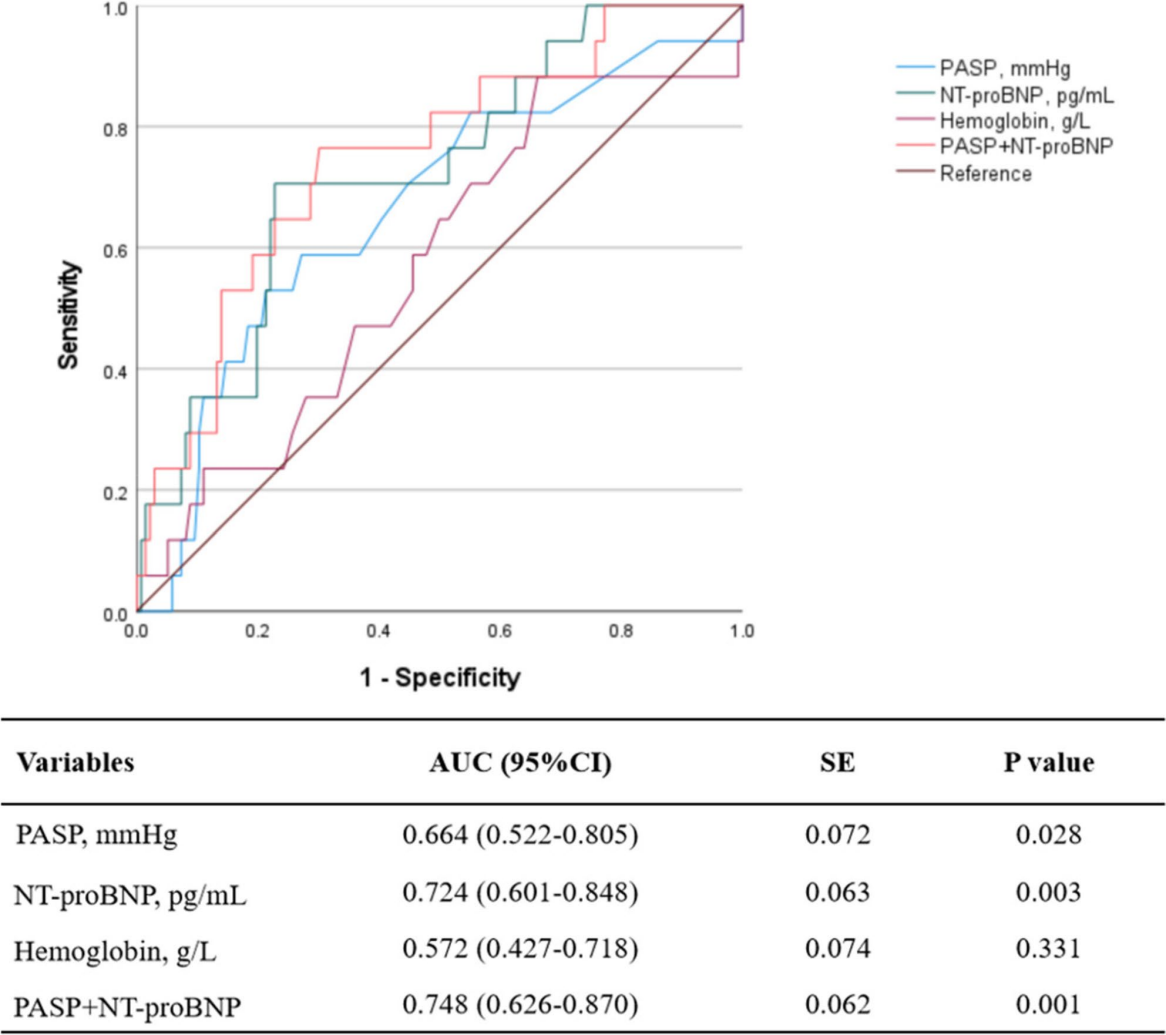
ROC curves for the predictive model discriminating CVD-related hospitalization or death based on different indicators. ROC, receiver operating characteristic; CVD, cardiovascular disease; AUC, area under curve; SE, standard error; PASP, pulmonary arterial systolic pressure; NT-proBNP, N-terminal-pro Brain natriuretic peptide

**Fig. 4:**
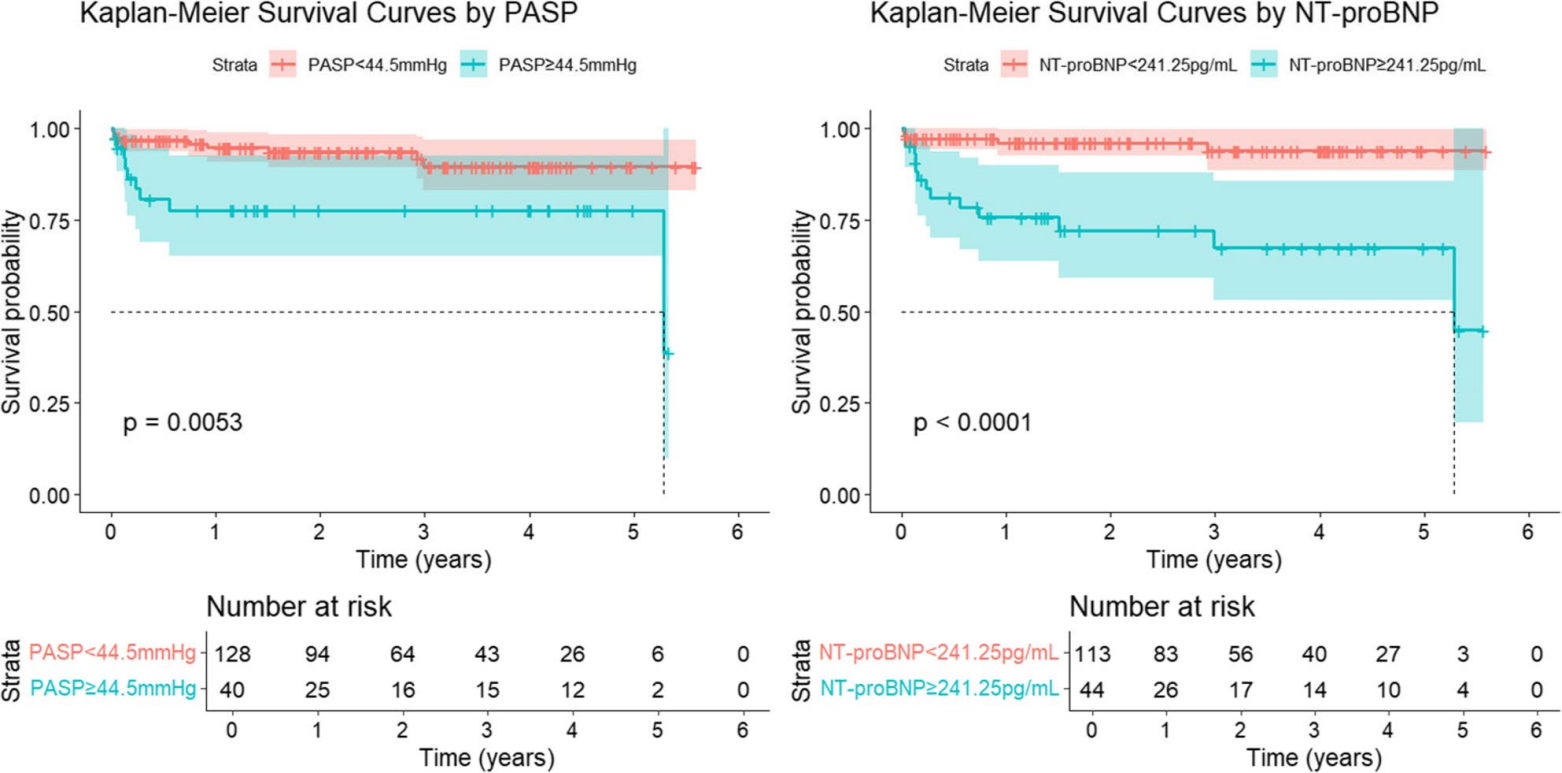
Kaplan-Meier survival curves for CVD-related hospitalization or death according to PASP and NT-proBNP. CVD, cardiovascular disease; PASP, pulmonary arterial systolic pressure; NT-proBNP, N-terminal-pro Brain natriuretic peptide

This study evaluated the predictive values of PASP, NT-proBNP, and Hb for cardiovascular hospitalizations and death events through ROC analysis. The VIF between PASP and NT-proBNP was 1.005, indicating no significant collinearity between these two variables. PASP demonstrated the highest predictive efficacy, with an area under the curve (AUC) of 0.664 (95% CI: 0.522–0.805, *P* = 0.028); the optimal cut-off value for PASP was determined as 44.5 mmHg (sensitivity 52.9%; specificity 79.6%). For NT-proBNP, the AUC was 0.724 (95% CI: 0.601–0.848, *P* = 0.003), with an optimal cut-off value of 241.25 pg/mL, yielding a sensitivity of 72.2% and a specificity of 77.7%. Notably, the combined PASP + NT-proBNP index achieved the most favorable predictive performance for the study endpoints, with an AUC of 0.748 (95% CI: 0.626–0.870, *P* = 0.001), a sensitivity of 76.5%, and a specificity of 70.1% (Fig. 3). When analyzed by stratified endpoints, the combination of PASP and NT-proBNP showed good predictive performance for CVD-related hospitalization (AUC = 0.818, *P* = 0.001), superior to any single marker; however, neither individual indicators nor the combined model showed significant predictive value for the endpoint of death (all *P* > 0.05) (Supplementary Table 5). Patients were further stratified into high-level and low-level subgroups according to the optimal cut-off values of PASP and NT-proBNP, respectively (Fig. 4). Survival analysis revealed that patients with lower PASP and NT-proBNP levels had significantly higher survival rates compared with those with elevated levels (log-rank test, *P* = 0.0053 and *P* < 0.0001, respectively). Specifically, patients with PASP ≥ 44.5mmHg had a significantly increased risk of CVD-related hospitalization or death during the follow-up period [hazard ratio (HR) = 3.456, 95% CI: 1.367–8.734, *P* = 0.009]. Similarly, patients with NT-proBNP ≥ 241.25 pg/mL demonstrated a markly elevated risk of the composite endpoint (HR = 6.863, 95% CI: 2.432–19.370, *P* < 0.001).

## Discussion

Pregnancy induces physiological adaptations in the maternal cardiovascular system. For women with pre-existing heart disease, suboptimal ventricular adaptation to these changes elevates the risk of adverse cardiovascular events [18, 19]. Moreover, maternal cardiac insufficiency impairs uteroplacental perfusion, which in turn contributes to unfavorable fetal outcomes [20, 21]. Consistent with previous studies [2, 22], our findings confirm the poor prognostic profile of pregnant women with severe CVD (mWHO Class III–IV). Sharma et al. [22] reported a maternal mortality rate of 5.45% in this group—significantly higher than the general maternal mortality rate of 1.12%—while the ROPAC study identified PH as the highest-risk subgroup with a mortality rate of 9% [2]. These findings reinforce the urgency of developing validated prognostic indicators and individualized management strategies to improve maternal-fetal outcomes in this high-risk cohort.

The present study confirmed the predictive value of NT-proBNP for adverse cardiovascular events (AUC = 0.724), which is highly consistent with the conclusions of several recent studies. In a retrospective study including 128 pregnant patients with heart disease, Chang et al. [23] found that an NT-proBNP level > 200 pg/mL was significantly associated with an increased risk of heart failure and preeclampsia. The cutoff value reported in their study was close to 241.25 pg/mL in the present study, confirming the clinical value of NT-proBNP as a general prognostic biomarker in this population. A study by Sharma et al. [22] in pregnant patients with pulmonary hypertension also demonstrated that elevated PASP was associated with increased maternal mortality. Although the PASP cutoff value they set (44 mmHg) was slightly lower than 44.5 mmHg in the present study, both support the validity of PASP as a prognostic indicator related to right heart function. Egbe et al. [24] have demonstrated that a temporal decline in right ventricular systolic function and RV-pulmonary artery coupling during pregnancy is independently associated with adverse outcomes in women with CHD. In addition, Dai et al. [25] identified NT-proBNP and PASP as important predictors of mortality risk in pregnant patients with pulmonary hypertension, which echoes the core indicator selection of our combined model and further validates the central role of these two indicators in prognostic assessment among high-risk populations.

Compared with most previous studies, the present study has two key distinctions. First, our study population focused on severe patients with mWHO class III–IV, whereas most prior studies included mild-to-moderate patients (mWHO class I–II) or mixed-risk populations [2, 22, 23]. Second, this study is the first to validate the incremental value of the combined PASP + NT-proBNP model in a severe population, whereas previous studies mainly focused on the independent predictive performance of single indicators. Notably, these differences do not contradict the conclusions of previous studies, but are results arising from heterogeneity in the study populations, and represent a complement to prior research.

The causes of such population heterogeneity can be explained from three aspects (Supplymentary Table 6). First, in terms of study population selection, over 90% of CHD patients in prior studies (e.g., Egbe et al. [24]) had mild cardiac insufficiency with good cardiovascular reserve, resulting in distinct baseline levels and change patterns of PASP and NT-proBNP compared to severe cases; in contrast, our enrolled patients had significantly impaired cardiac function and heavier hemodynamic load, potentially amplifying the two indicators’ combined predictive efficacy. Second, differences in sample size and disease subtype composition are relevant: our study included multiple subtypes (CHD 58.3%, VHD 2.8%, PH 25.0%), whereas most prior research focused on a single type (e.g., isolated CHD [24]). Distinct pathophysiological characteristics of each subtype (e.g., cardiomyopathy elevating NT-proBNP [26], VHD interfering with PASP assessment [27], CHD involves chronic anatomical abnormalities with unique prognostic mechanisms [28]) may affect predictive performance, and our limited sample size—compared to larger multicenter studies (e.g., Dai et al. [25])—may further contribute to divergent results. Third, study design and endpoint definition differ: our follow-up identified 18 composite endpoint events (9 hospitalizations, 9 deaths), which improved statistical power by combining rare and common events, whereas some prior studies used a single endpoint (e.g., death [25] or HF [23]), reducing comparability. Additionally, while we controlled for confounding factors (e.g., gestational age, basic medication), residual bias—absent in some prior studies—may serve as a secondary cause of differences.

Notably, the combined PASP + NT-proBNP index yielded the highest predictive performance among all tested indicators (AUC = 0.748, *P* = 0.001), representing a modest but clinically meaningful improvement over NT-proBNP alone. This incremental value may be attributed to the fact that PASP and NT-proBNP reflect distinct pathophysiological aspects of cardiovascular dysfunction: PASP directly assesses RV afterload and pulmonary vascular status, while NT-proBNP reflects global cardiac stress and ventricular dysfunction. Their combination thus captures multiple dimensions of cardiovascular pathophysiology that neither marker alone can fully reflect, enhancing the comprehensiveness of prognostic assessment. Similar to our study, the PREG-CVD-HH study also focused on pregnant women with cardiovascular disease and consistently found that cardiac function temporarily deteriorates in the third trimester and perinatal period, with fluctuations in related biomarkers that gradually recover postpartum; however, the PREG-CVD-HH study included more biomarkers (such as NT-proBNP, mid-regional pro-adrenomedullin (MRproADM) and high-sensitivity cardiac troponin I (hs-cTnI)) and echocardiographic indicators, focusing on observing the natural evolution trajectory of perinatal cardiac function [29]. From a clinical perspective, this improved discrimination may translate into more precise risk stratification—for example, identifying high-risk patients who may benefit from intensified monitoring (e.g., more frequent echocardiographic assessments, serial NT-proBNP measurements) or targeted interventions (e.g., early initiation of pulmonary vasodilators, optimized heart failure therapy) during pregnancy and the postpartum period. However, it is important to acknowledge that the clinical utility of this combined model requires further validation in larger, prospective cohorts to confirm its ability to influence clinical decision-making and improve patient outcomes. Our combined model aligns with Dai et al. [25], who identified NT-proBNP, PASP, and albumin as the most significant predictors of mortality in pregnant women with PH, but extends to a broader severe CVD cohort and composite endpoint.

Anemia is a well-recognized major risk factor that worsens prognostic outcomes in pregnancy, as it reduces oxygen-carrying capacity, impairs maternal-fetal oxygenation, and exacerbates cardiac hemodynamic burden in the context of pregnancy-induced high cardiac output [30, 31]. Physiologically, maternal Hb concentration declines during pregnancy, reaching a nadir in the third trimester [32]. Our study extends this observation to women with severe CVD, demonstrating that Hb levels were significantly lower in women who achieved successful delivery compared to those who experienced abortion (10.6 g/L vs. 11.8 g/L, *P* = 0.000; Supplementary Table 1). Concomitantly, the proportion of patients receiving iron supplementation was substantially higher in the successful delivery group (30.2% vs. 6.1%, *P* = 0.001), suggesting that targeted iron supplementation may improve prognostic outcomes by mitigating anemia-related hemodynamic burden. However, our data also highlight the complexity of Hb management in this population: multivariate analysis identified Hb ≥ 11.1 g/dL as an independent negative predictor of successful delivery. This exploratory association requires cautious interpretation, as Hb levels in this population are co-modulated by disease-related pathologies (e.g., hemoconcentration, cardiac insufficiency) and pregnancy-specific physiological adaptations (e.g., increased blood volume) [33]. No direct causal relationship is confirmed [34, 35]; and Hb cannot serve as an independent prognostic indicator for delivery outcomes, and further validation is required through large-scale studies.

Delivery mode selection is a critical clinical decision in managing pregnant women with severe CVD, given the heightened maternal and neonatal risk in this population—evidenced by Owens et al. [36] who reported 16.1% major adverse cardiac events (MACE) among 3,871 women with heart disease (highest in cardiomyopathy: 45.9%, PH: 25%) and increased neonatal complications, particularly with maternal cardiomyopathy or PH. Consistent with our earlier prognostic findings for PASP (PASP ≥ 50 mmHg predicts poor perinatal outcomes and long-term risk; PASP ≥ 44.5 mmHg linked to increased hospitalization / death risk), our cohort had a notably high cesarean section rate (83.7%)—starkly contrasting with the 55.0% cesarean section rate in the general obstetric population reported by Guner et al. [37]. This discrepancy stems from distinct clinical priorities: whereas prior uterine surgery and fetal distress are the primary drivers of cesarean section in the general obstetric population [37], mitigating labor-induced cardiac decompensation takes precedence in managing women with severe CVD.

In summary, this preliminary exploratory study draws the following valuable conclusions: (1) A PASP ≥ 50 mmHg is associated with poor perinatal outcomes in pregnant women with severe CVD; (2) A PASP ≥ 44.5 mmHg and an NT-proBNP ≥ 241.25 pg/mL can stratify the risk of the composite endpoint (CVD-related hospitalization or death), with these cutoff values being exploratory and not definitive clinical thresholds; (3) Combined PASP + NT-proBNP detection offers superior prognostic efficacy, supporting a tailored clinical strategy: PASP for perinatal prognostic assessment and NT-proBNP for long-term cardiovascular risk stratification. This strategy may optimize management of this high-risk cohort. To enhance clinical applicability, we propose rounding cut-offs to PASP 45 mmHg and NT-proBNP 240 pg/mL. Although this adjustment may slightly compromise sensitivity and specificity, it can substantially improve the convenience of routine clinical practice and is more in line with actual clinical needs. It is important to emphasize that the cut-off values identified in this study are exploratory and hypothesis-generating, rather than definitive thresholds for clinical decision-making. Given the single-center, retrospective nature of the study, these cut-offs may not be generalizable to other populations and require further validation. Critically, while the findings provide important clinical references for the management of high-risk pregnant women with mWHO Class III–IV cardiovascular disease, they cannot be directly generalized to broader populations or lower-risk pregnant groups.

This study has several limitations. First, the study adopted a single-center design with a relatively small sample size, which not only reduces the statistical power of the analysis but also introduces inherent sample selection bias. The study population was limited to patients from a single institution, lacking representativeness of diverse clinical settings (e.g., different levels of hospitals, regional medical resources). This selection bias may distort the observed correlations between PASP/NT-proBNP levels and adverse pregnancy outcomes—for instance, the study population may over-represent patients with severe disease or specific clinical characteristics, leading to overestimation or underestimation of the actual association strength. Additionally, the small sample size, limited number of events, and individual differences in the CVD subgroup increase the risk of overfitting of ROC cutoffs, which may reduce the reliability of the determined PASP and NT-proBNP cutoffs in clinical application. Furthermore, no external validation was performed for the identified cut-offs or the combined predictive model, so their generalizability and stability in different populations remain unconfirmed. This is a key limitation that highlights the need for future multicenter prospective studies to validate these findings before they can be incorporated into routine clinical practice. Second, the study period overlapped with the Coronavirus Disease 2019 (COVID-19) pandemic; unmeasured confounders such as Severe Acute Respiratory Syndrome Coronavirus 2 (SARS-CoV-2) infection (which may exacerbate pulmonary vasculopathy, coagulopathy, or myocardial dysfunction) and vaccination status could have influenced thrombotic, pulmonary, and overall maternal outcomes—particularly in patients with pre-existing PH or ventricular dysfunction. Third, diagnostic and measurement limitations exist. The diagnosis of PH relied solely on echocardiography (invasive RHC was unavailable), potentially affecting diagnostic accuracy. PASP was estimated by echocardiography, and its results were prone to variability affected by the quality of tricuspid regurgitation signals, the operator’s measurement experience, and individual differences in cardiac anatomy. Moreover, compared with RHC, echocardiography had certain limitations in terms of accuracy, especially in patients with insufficient tricuspid regurgitation signals, severe tricuspid regurgitation with turbulent flow, or complex cardiac structural abnormalities. Fourth, incomplete documentation of key obstetric variables precluded consistent application of the Robson 10-group classification for cesarean section benchmarking, while telephone follow-up restricted fetal outcome monitoring and only 10% of participants had complete serum ferritin data, limiting assessment of maternal iron status and its interaction with hemoglobin levels. Collectively, these limitations underscore that the findings of this study should be interpreted with caution. Clinical decision-making for pregnant patients with PH should integrate the present data with individual patient characteristics, comprehensive clinical assessments, and multidisciplinary team evaluations.

To address this gap, future prospective studies should address these limitations by adopting multi-center designs, larger sample sizes, and standardized data collection (including COVID-19-related variables, predefined obstetric parameters, and in-person follow-up). Additionally, exploring associations between speckle-tracking echocardiographic parameters, pregnancy outcomes, and long-term cardiovascular events in this high-risk cohort could help develop a more holistic risk assessment system, providing actionable evidence for clinical decision-making [38, 39].

## Conclusions

PASP may serve as a core monitoring indicator for perinatal risk assessment in pregnant women with CVD, while NT-proBNP could act as a critical adjunct for refining the prediction of long-term cardiovascular events. The combined use of these two parameters may help optimize risk stratification, thereby providing preliminary guidance for perinatal management and long-term prognosis monitoring in this specific patient cohort.

## Supplementary Information

Supplementary Material 1.

Supplementary Material 2.

## Data Availability

The datasets used in this study are available from the corresponding author on reasonable request.

## Abbreviations

ASE: American Society of Echocardiography
AUC: Area under the curve
CAD: Coronary heart disease
CARPREG: Cardiac Disease in Pregnancy
CHD: Congenital heart disease
CI: Confidence interval
COVID-19: Coronavirus Disease 2019
CVD: Cardiovascular disease
DCM: Dilated cardiomyopathy
EMR: Electronic medical record
Hb: Hemoglobin
HCM: Hypertrophic cardiomyopathy
HDP: Hypertensive disorders of pregnancy
HF: Heart failure
hs-CRP: High-sensitivity C-reactive protein
hs-cTnI: High-sensitivity cardiac troponin I
LVEF: Left ventricular ejection fraction
MDT: Multidisciplinary teams
MMR: Maternal mortality ratio
MRproADM: Mid-regional pro-adrenomedullin
mWHO: modified WHO
NT-proBNP: N-terminal-pro Brain natriuretic peptide
NYHA: New York Heart Association
OR: Odds ratio
PASP: Pulmonary arterial systolic pressure
PH: Pulmonary hypertension
RAP: Right atrial pressure
ROC: Receiver operating characteristic
ROPAC: Registry of Pregnancy and Cardiology
RV: Right ventricular
RVOT: Right ventricular outflow tract
SARS-CoV-2: Severe Acute Respiratory Syndrome Coronavirus 2
SD: Standard deviation
SDGs: Sustainable Development Goals
TRV: Tricuspid regurgitation jet velocity
UA: Uric acid
VHD: Valvular heart disease
VIF: Variance inflation factor
ZAHARA: Zwangerschap bij Aangeboren HARtAfwijkingen
