# Supplementary material for: Predictive efficacy of PASP combined with NT-proBNP for outcomes in pregnant women with severe cardiovascular disease: a single-centre retrospective observational study
Source: BMC Pregnancy Childbirth. 2026 May 22;26:772. doi: 10.1186/s12884-026-09288-7 (PMC13371250; doi:10.1186/s12884-026-09288-7)
Supplement: Supplementary file 1 — Supplementary Material 1. [file 12884_2026_9288_MOESM1_ESM.docx]

**Supplymentary Table 1. Baseline characteristics, management, and outcomes of pregnant women with severe cardiovascular disease**

| **Variables** | **Total**  **N = 168** | **Abortion**  **N = 82** | **Delivery**  **N = 86** | **P value** |
| --- | --- | --- | --- | --- |
| **Demographic Information** |  |  |  |  |
| Age, years | 31.0 (6.0) | 32.0 (7.0) | 30.0 (4.0) | 0.059 |
| Nulliparous, n (%) | 61 (36.3) | 43 (52.4) | 18 (20.9) | 0.000 |
| History of Abortion, n (%) |  |  |  |  |
| 0 | 95 (56.5) | 35 (42.7) | 60 (69.8) | 0.001 |
| 1 | 44 (26.2) | 24 (29.3) | 20 (23.2) |  |
| 2 | 18 (10.7) | 16 (19.5) | 2 (2.3) |  |
| 3 | 5 (3.0) | 3 (3.7) | 2 (2.3) |  |
| 4 | 6 (3.6) | 4 (4.9) | 2 (2.3) |  |
| Child, n (%) |  |  |  |  |
| 0 | 110 (65.5) | 42 (51.2) | 68 (79.0) | 0.003 |
| 1 | 44 (26.2) | 32 (39.0) | 12 (14.0) |  |
| 2 | 11 (6.5) | 6 (7.3) | 5 (5.8) |  |
| 3 | 2 (1.2) | 1 (1.2) | 1 (1.2) |  |
| 4 | 1 (0.6) | 1 (1.2) | 0 (0.0) |  |
| **Length of stay, days** | 6.0 (3.0) | 5.0 (5.0) | 6.5 (4.0) | 0.000 |
| **Diagnosis made** |  |  |  |  |
| Before pregnancy, n (%) | 121 (72.0) | 64 (78.0) | 57 (66.3) | 0.089 |
| During pregnancy, n (%) | 47 (28.0) | 18 (22.0) | 29 (33.7) |  |
| **Diagnosis** |  |  |  |  |
| CHD, n (%) | 98 (58.3) | 43 (52.4) | 55 (64.0) | 0.130 |
| No surgery, n (%) | 51 (52.0) | 22 (51.2) | 29 (52.7) | 0.878 |
| Surgical intervention, n (%) | 47 (48.0) | 21 (48.8) | 26 (47.3) |  |
| VHD, n (%) | 72 (42.9) | 44 (53.7) | 28 (32.6) | 0.006 |
| No surgery, n (%) | 30 (41.7) | 14 (31.8) | 16 (57.1) | 0.034 |
| Surgical intervention, n (%) | 42 (58.3) | 30 (68.2) | 12 (42.9) |  |
| Aortic dissection, n (%) | 5 (3.0) | 2 (2.4) | 3 (3.5) | 1.000 |
| Arrhythmia, n (%) | 11 (6.5) | 3 (3.6) | 8 (9.3) | 0.212 |
| PH, n (%) | 42 (25.0) | 21 (25.6) | 21 (24.4) | 0.859 |
| HCM/DCM, n (%) | 8 (4.8) | 4 (4.9) | 4 (4.7) | 1.000 |
| Hypertension, n (%) | 7 (4.2) | 2 (2.4) | 5 (5.8) | 0.444 |
| HF, n (%) | 5 (3.0) | 2 (2.4) | 3 (3.5) | 1.000 |
| CAD, n (%) | 3 (1.8) | 1 (1.2) | 2 (2.3) | 1.000 |
| Endocarditis or myocarditis, n (%) | 3 (1.8) | 2 (2.4) | 1 (1.2) | 0.614 |
| **NYHA class and echocardiography** |  |  |  |  |
| I, n (%) | 90 (53.6) | 40 (48.8) | 50 (58.1) | 0.411 |
| II, n (%) | 62 (36.9) | 33 (40.2) | 29 (33.7) |  |
| III, n (%) | 15 (8.9) | 9 (11.0) | 6 (7.0) |  |
| IV, n (%) | 1 (0.6) | 0 (0.0) | 1 (1.2) |  |
| PASP, mmHg | 34.0 (14.0) | 36.0 (17.0) | 34.0 (11.0) | 0.048 |
| LVEF, % | 65.0 (5.0) | 66.0 (4.0) | 64.0 (6.0) | 0.013 |
| **Laboratory examination** |  |  |  |  |
| Hemoglobin, g/L | 111.0 (21.0) | 118.0 (23.0) | 106.0 (18.0) | 0.000 |
| NT-proBNP, pg/ml | 131.5 (188.3) | 156.0 (153.5) | 108.0 (207.7) | 0.009 |
| D-dimer, mg/L | 1.42 (2.65) | 0.42 (0.69) | 2.42 (2.51) | 0.000 |
| hs-CRP, mg/L | 36.2 (46.58) | 5.0 (10.75) | 42.6 (31.5) | 0.000 |
| UA, µmol/L | 263.5 (96.0) | 238.0 (76.0) | 296.0 (91.25) | 0.000 |
| Creatinine, µmol/L | 50.0 (10.0) | 48.5 (10.5) | 51.0 (12.75) | 0.014 |
| **Gestational Week** |  |  |  |  |
| Q ≤ 20 weeks, n (%) | 80 (47.6) | 78 (95.1) | 2 (2.3) | 0.000 |
| 20 < Q ≤ 30 weeks, n (%) | 5 (3.0) | 4 (4.9) | 1 (1.2) |  |
| 30 < Q ≤ 35 weeks, n (%) | 9 (5.4) | 0 (0.0) | 9 (10.5) |  |
| Q > 35 weeks, n (%) | 74 (44.0) | 0 (0.0) | 74 (86.0) |  |
| **Pregnancy outcome** |  |  |  |  |
| Surgical abortion, n (%) | 55 (32.7) | 55 (67.1) | 0 (0.0) | 0.000 |
| Drug abortion, n (%) | 27 (16.1) | 27 (32.9) | 0 (0.0) |  |
| Vaginal delivery, n (%) | 14 (8.3) | 0 (0.0) | 14 (16.3) |  |
| Cesarean section, n (%) | 72 (42.9) | 0 (0.0) | 72 (83.7) |  |
| **Anesthesia** |  |  |  |  |
| No, n (%) | 39 (23.2) | 26 (31.7) | 13 (15.1) | 0.000 |
| Epidural anesthesia, n (%) | 71 (42.3) | 0 (0.0) | 71 (82.6) |  |
| General anesthesia, n (%) | 58 (34.5) | 56 (68.3) | 2 (2.3) |  |
| **Arrested or Suspended embryo growth** | | | | |
| Yes, n (%) | 24 (14.3) | 14 (17.1) | 10 (11.6) | 0.313 |
| No, n (%) | 144 (85.7) | 68 (82.9) | 76 (88.3) |  |
| **Medications after discharge** | | | | |
| Anticoagulants | 82 (48.8) | 22 (26.8) | 60 (69.8) | 0.000 |
| Antihypertensive drugs | 10 (6.0) | 2 (2.4) | 8 (9.3) | 0.060 |
| Loop diuretics | 14 (8.3) | 5 (6.1) | 9 (10.5) | 0.306 |
| Spironolactone | 7 (4.2) | 4 (4.9) | 3 (3.5) | 0.652 |
| Warfarin | 9 (5.4) | 7 (8.5) | 2 (2.3) | 0.074 |
| Iron supplement | 31 (18.5) | 5 (6.1) | 26 (30.2) | 0.000 |
| **PAH target drugs** |  |  |  |  |
| Monotherapy | 2 (1.2) | 0 (0.0) | 2 (2.3) | 0.154 |
| Dual-agent Therapy | 9 (5.4) | 7 (8.5) | 2 (2.3) |  |
| Triple-agent Therapy | 3 (1.8) | 1 (1.2) | 2 (2.3) |  |

Values are presented as median (interquartile range) or n (%). CHD, congenital heart disease; VHD, valvular heart disease; PH, pulmonary hypertension; HCM, hypertrophic cardiomyopathy; DCM, dilated cardiomyopathy; HF, heart failure; CAD, coronary heart disease; NYHA, New York Heart Association; PASP, pulmonary arterial systolic pressure; NT-proBNP, N-terminal-pro Brain natriuretic peptide; LVEF, left ventricular ejection fraction; PAH, pulmonary arterial hypertension.

**Supplymentary Table 2. Characteristics of patients with CVD-related hospitalization**

| **Characters** | **Case1** | **Case2** | **Case3** | **Case4** | **Case5** | **Case6** | **Case7** | **Case8** | **Case9** |
| --- | --- | --- | --- | --- | --- | --- | --- | --- | --- |
| Age years | 22 | 25 | 29 | 33 | 34 | 43 | 32 | 18 | 30 |
| Parity | G2P0 | G1P0 | G1P0 | G2P1 | G1P0 | G1P0 | G1P0 | G1P0 | G2P1 |
| Etiology of disease | CHD, severe PH, Eisenmenger syndrome | ASD+PH | ASD | VHD | CHD+VHD | VHD | VSD+severe PH, Eisenmenger syndrome | Aortic dissection | VHD+PH |
| Arrested or Suspended embryo growth | No | No | No | No | No | No | No | No | No |
| NYHA class | 3 | 2 | 1 | 2 | 2 | 1 | 4 | 2 | 1 |
| PASP, mmHg | 72 | 71 | 42 | 45 | 33 | 54 | 82 | 30 | 73 |
| LVEF, % | 65 | 66 | 67 | 62 | 55 | 69 | 38 | 69 | 65 |
| NT-proBNP, pg/mL | 86.1 | 262.7 | 244.4 | 275 | 2240 | 299.8 | 4657 | / | 250.5 |
| Hemoglobin,g/dL | 117 | 140 | / | 104 | / | 103 | 101 | 113 | 135 |
| Termination of G. weeks | 35.6 | 13.6 | 9.1 | 16.4 | 5.4 | 35.7 | 37.0 | / | 8.6 |
| Delivery model | cesarean delivery | medical abortion | surgical abortion | surgical abortion | surgical abortion | cesarean delivery | cesarean delivery | cesarean delivery | surgical abortion |
| Anesthesia model | epidural anesthesia | No | general anesthesia | general anesthesia | general anesthesia | epidural anesthesia | epidural anesthesia | epidural anesthesia | general anesthesia |
| Fetal weight, g | / | / | / | / | / | 2265 | / | / | / |
| Follow-up PASP, mmHg | 87 | 35 | 24 | 31 | 35 | 26 | 87 | 30 | 30 |
| Follow-up LVEF, % | 65 | 68 | 67 | 70 | 62 | 59 | 37 | 65 | 64 |
| Timing death, years | 0.23 | 0.12 | 0.13 | 0.27 | 1.50 | 0.55 | 5.29 | 0.92 | 0.15 |
| Reason for readmission | Worsening of HF | Repair of ASD | Repair of ASD | Percutaneous balloon mitral valvuloplasty | 3° Atrioventricular block, pacemaker implantation | Mechanical valve replacement | Worsening of HF | Aortic replacement | Percutaneous balloon mitral valvuloplasty |

CVD, cardiovascular disease; CHD, congenital heart disease; VHD, valvular heart disease; PH, pulmonary hypertension; ASD, atrial septal defect; VSD, ventricular septal defect; HF, heart failure; NYHA, New York Heart Association; NT-proBNP, N-terminal-pro Brain natriuretic peptide; PASP, pulmonary arterial systolic pressure; LVEF, left ventricular ejection fraction.

**Supplymentary Table 3. Characteristics of maternal mortalities**

| **Characters** | **Case1** | **Case2** | **Case3** | **Case4** | **Case5** | **Case6** | **Case7** | **Case8** | **Case9** |
| --- | --- | --- | --- | --- | --- | --- | --- | --- | --- |
| Age years | 26 | 34 | 26 | 23 | 32 | 33 | 32 | 38 | 35 |
| Parity | G2P0 | G4P1 | G2P0 | G1P0 | G6P4 | G2P0 | G1P0 | G8P4 | G5P1 |
| Etiology of disease | mechanical aortic valve replacement | VSD repair combined with infective endocardit | TOF | TOF (after surgery) | type B aortic dissection | TOF | severe pulmonary valve stenosis | type A aortic dissection (after surgery) | mechanical mitral valve replacement |
| Complication |  |  |  |  | Hypertension, diabetes | Hypertension, proteinuria |  |  |  |
| Arrested or Suspended embryo growth | Yes | No | Yes | No | No | No | No | No | Yes |
| NYHA class | 1 | 2 | 3 | 1 | 1 | 3 | 1 | 2 | 2 |
| PASP, mmHg | 22 | 36 | 92 | 47 | 34 | / | 73 | 30 | 35 |
| LVEF, % | 66 | 69 | 62 | 65 | 60 | 58 | 71 | 65 | 64 |
| NT-proBNP, pg/mL | 82 | 563 | 95 | 108 | 121.8 | 895 | 249.5 | 54.7 | 255 |
| Hemoglobin,g/dL | 117 | 106 | 205 | / | 79 | 224 | 111 | 109 | 114 |
| Termination of G. weeks | 10.1 | 11.3 | 17.1 | 7.3 | 37.4 | 9.4 | 38.0 | 15.4 | 7.3 |
| Delivery model | spontaneous abortion | medical abortion | medical abortion | surgical abortion | cesarean delivery | surgical abortion | cesarean delivery | medical abortion | surgical abortion |
| Anesthesia model | No | No | No | general anesthesia | epidural anesthesia | general anesthesia | epidural anesthesia | general anesthesia | general anesthesia |
| Fetal weight, g | / | / | / | / | 2735 | / | 2960 | / | / |
| Follow-up PASP, mmHg | 35 | 35 | 85 | 47 | 34 | / | 73 | 30 | / |
| Follow-up LVEF, % | 65 | 64 | 62 | 65 | 60 | 58 | 71 | 65 | / |
| Timing death, years | 2.93 | 0.73 | 0.02 | 0.05 | 0.01 | 2.98 | 0.13 | 0.03 | 0.02 |
| CardiogenicDeath | Yes | Yes | Yes | Yes | Yes | Yes | Yes | No | Yes |

These 9 fatal cases included 3 cases of tetralogy of Fallot (TOF) (1 case underwent surgery, 1 case after mechanical aortic valve replacement, 1 case after mechanical mitral valve replacement, 1 case of ventricular septal defect (VSD) repair combined with infective endocarditis, 1 case of severe pulmonary valve stenosis, 1 case of type B aortic dissection, and 1 case of type A aortic dissection (who underwent David procedure + total aortic arch artificial vessel replacement with stented elephant trunk surgery). VSD, ventricular septal defect; TOF, tetralogy of Fallot; NYHA, New York Heart Association; NT-proBNP, N-terminal-pro Brain natriuretic peptide; PASP, pulmonary arterial systolic pressure; LVEF, left ventricular ejection fraction.

**Supplymentary Table 4. The follow-up situation of PASP in women with severe cardiovascular complications under different comorbidities and pregnancy outcomes**

| Variables | PASP before discharge | Follow-up of PASP | P value |
| --- | --- | --- | --- |
| **PASP, mmHg** |  |  |  |
| PASP ≥ 50mmHg | 79.0 (28.0)^****^ | 72.0 (42.0)^****^ | 0.039 |
| PASP ＜ 50mmHg | 32.0 (8.0) | 32.0 (6.0) | 0.079 |
| **Diagnosis** |  |  |  |
| CHD | 35.0 (17.0)^ns^ | 34.0 (16.0)^ns^ | 0.0062 |
| Non-CHD | 33.0 (12.0) | 34.5 (9.0) | 0.202 |
| **Diagnosis** |  |  |  |
| VHD | 34.0 (9.0)^ns^ | 35.0 (7.0)^ns^ | 0.152 |
| Non-VHD | 35.0 (19.0) | 34.0 (17.0) | 0.0195 |
| **Diagnosis** |  |  |  |
| PH | 63.5 (38.0)^****^ | 45.0 (46.0)^****^ | 0.0054 |
| Non-PH | 32.0 (6.0) | 32.0 (6.0) | 0.3109 |
| **Pregnancy outcome** |  |  |  |
| Delivery | 34.0 (11.0)^*^ | 33.0 (8.0)^ns^ | 0.0956 |
| Abortion | 36.0 (17.0) | 35.0 (14.0) | 0.0264 |

Values are presented as median (interquartile range) or n (%). PASP, pulmonary arterial systolic pressure; CHD, congenital heart disease; VHD, valvular heart disease; PH, pulmonary hypertension; ns, not significant; *P < 0.05, **P < 0.01, ***P < 0.001, ****P < 0.0001.

**Supplymentary Table 5. Predictive Performance of Main Evaluation Indicators for CVD-related hospitalization and Death as Separate Endpoints**

| **Variables** | **AUC (95%CI）** | **SE** | **P value** |
| --- | --- | --- | --- |
| **CVD-related hospitalization** |  |  |  |
| PASP, mmHg | 0.741 (0.584-0.899) | 0.080 | 0.015 |
| NT-proBNP, pg/mL | 0.794 (0.649-0.939) | 0.074 | 0.003 |
| Hemoglobin, g/L | 0.575 (0.381-0.770) | 0.099 | 0.448 |
| PASP+NT-proBNP | 0.818 (0.670-0.967) | 0.076 | 0.001 |
| **Death** |  |  |  |
| PASP, mmHg | 0.563 (0.344-0.782) | 0.112 | 0.549 |
| NT-proBNP, pg/mL | 0.654 (0.479-0.828) | 0.089 | 0.123 |
| Hemoglobin, g/L | 0.605 (0.403-0.807) | 0.103 | 0.292 |
| PASP+NT-proBNP | 0.641 (0.468-0.814) | 0.088 | 0.180 |

AUC, area under curve; CI, confidence interval; SE, standard error; CVD, cardiovascular disease; PASP, pulmonary arterial systolic pressure; NT-proBNP, N-terminal-pro Brain natriuretic peptide.

**Supplymentary Table 6. The predictive efficacy of combined PASP + NT-proBNP index in pregnant women with different comorbidity subgroups**

| **Variables** | **AUC (95%CI）** | **SE** | **P value** |
| --- | --- | --- | --- |
| **Subgroup** |  |  |  |
| PH (n=42) | 0.737 (0.558-0.916) | 0.091 | 0.067 |
| Non-PH (n=126) | 0.734 (0.575-0.893) | 0.081 | 0.011 |
| **Diagnosis** |  |  |  |
| CHD (n=98) | 0.869 (0.777-0.960) | 0.047 | 0.000 |
| VHD (n=72) | 0.852 (0.678-1.000) | 0.089 | 0.001 |
| Non-CHD/VHD (n=26) | 0.208 (0.046-0.371) | 0.083 | 0.178 |

AUC, area under curve; CI, confidence interval; SE, standard error; PH, pulmonary hypertension; CHD, congenital heart disease; VHD, valvular heart disease.
