# Supplementary material for: Predictive efficacy of PASP combined with NT-proBNP for outcomes in pregnant women with severe cardiovascular disease: a single-centre retrospective observational study
Source: BMC Pregnancy Childbirth. 2026 May 22;26:772. doi: 10.1186/s12884-026-09288-7 (PMC13371250; doi:10.1186/s12884-026-09288-7)
Supplement: Supplementary file 2 — Supplementary Material 2. [file 12884_2026_9288_MOESM2_ESM.pdf]

SPRINGER NATURE

Change of authorship request form - Journals (pre-acceptance)

Important information. Please read.

- This form should be used by authors to request any change in authorship (adding/deleting authors) including changes in corresponding authors. This form should not be used for name changes. Please fully complete all sections. Use black ink and block capitals and provide each author's full name with the given name first followed by the family name.

- By signing this declaration, all authors guarantee that the order of the authors are in accordance with their scientific contribution, if applicable as different conventions apply per discipline, and that only authors have been added who made a meaningful contribution to the work.

- Please note, in author collaborations where there is formal agreement for representing the collaboration, it is sufficient for the representative or legal guarantor (usually the corresponding author) to complete and sign the Authorship Change Form on behalf of all authors, **next to the added/removed author(s). (Complete Section 3, followed by Section 6.)**
  In author collaborations where there is no formal agreement for representing the collaboration and **there are more than 10 authors**, one may sign for all, provided the signer appends correspondence that attests that each of the authors have agreed to the change **and the added/removed authors sign the form. (Complete Section 3, followed by Section 6.)**

- Please note, we cannot investigate or mediate any authorship disputes. If you are unable to obtain agreement from all authors (including those who you wish to be removed) you must refer the matter to your institution(s) for investigation. Please inform us if you need to do this.

- If you are not able to return a fully completed form within **30 days** of the date that it was sent to the author requesting the change, we may have to withdraw your manuscript. We cannot publish manuscripts where authorship has not been agreed by all authors (including those who have been removed).

- Incomplete forms will be rejected.

- Please return/upload this form, fully completed, to the Journals Editorial Office. The Journal and/or Publisher will consider the information you have provided to decide whether to approve the proposed change in authorship. We may decide to contact your institution for more information or undertake a further investigation, if appropriate, before making a final decision.

SPRINGER NATURE   Change of authorship request form - Journals (pre-acceptance)

Section 1: Please provide the current title of manuscript

**Manuscript ID no.:**	d72cab09-460c-45e8-ad67-bdc67e04ebf0 v2.0

Title:	Predictive efficacy of PASP combined with NT-proBNP for outcomes in pregnant women with severe cardiovascular disease: a single-centre retrospective observational study

Section 2: Please provide the previous authorship, in the order shown on the manuscript before the changes were introduced. Please indicate the corresponding author by adding (CA) behind the name.

	First name(s)	Family name	ORCID or SCOPUS id, if available
1st author	Dan	Tian	
2nd author	Dandan	chen	
3rd author	Min	Tang	
4th author	Dawei	Lin	
5th author	Qi	Jin	
6th author	Fan	Yang	
7th author	Yang	Zhan	
8th author	Daxin	Zhou	
9th author	Jiarong	Zhang (CA)	
10th author	Qianzhou	Lv (CA)	

Please use an additional sheet if there are more than 10 authors.

11th author	Lihua	Guan (CA)	
12th author	Junbo	Ge	
13th author			

SPRINGER NATURE Springer Nature is one of the world's leading global research, educational and professional publishers, created in May 2015 through the combination of Nature Publishing Group, Palgrave Macmillan, Macmillan Education and Springer Science+Business Media.

SPRINGER NATURE Change of authorship request form - Journals (pre-acceptance)

Section 3: Please provide a justification for change. Please use this section to explain your reasons for changing the authorship of your manuscript, e.g. what necessitated the change in authorship? Please refer to the (journal) policy pages for more information about authorship. Please explain why omitted authors were not originally included and/or why authors were removed on the submitted manuscript.

We propose to add Professor Dehong Kong from the Department of Echocardiography as an additional co-corresponding author. This adjustment is justified by Professor Kong's significant intellectual guidance and practical contributions to the development of the core conclusions of this study.
Specifically, the key dataset forms the foundation of the core conclusions of this research, and Professor Kong was directly responsible for the quality control and provenance verification of this dataset. She led the measurement of core parameters derived from echocardiography (including pulmonary artery systolic pressure), formulated and implemented rigorous quality assurance protocols, and is the sole responsible party for this core data. Given that this dataset underpins the analytical reasoning and core conclusions of the study, we believe that appointing Professor Kong as a co-corresponding author—who will address future inquiries regarding these core methodological aspects—is not only academically compliant but also serves to clarify accountability.
All authors of this study have been consulted regarding this adjustment and have unanimously approved it

Section 4: Proposed new authorship. Please provide your new authorship list in the order you would like it to appear on the manuscript. Please indicate the corresponding author by adding (CA) behind the name. <u>If the Corresponding Author has changed, please indicate the reason under section 3</u>.

	First name(s)	Family name (this name will appear in full on the final publication and will be searchable in various abstract and indexing databases)	Affiliated institute	E-mail address
1st author	Dan	Tian	Zhongshan Hospital Fudan University	tian.dan@zs-hospital.sh.cn
2nd author	Dandan	Chen	Zhongshan Hospital Fudan University	chen.dandan@zs-hospital.sh.cn
3rd author	Min	Tang		tang.min@zs-hospital.sh.cn
4th author	Dawei	Lin		lindaweiii@163.com
5th author	Qi	Jin	Zhongshan Hospital Fudan University	jin.qi@zs-hospital.sh.cn
6th author	Fan	Yang	Shanghai Geriatric Medical center	fanyang3229@163.com
7th author	Yang	Zhan	Zhongshan Hospital Fudan University	zhan.yang@zs-hospital.sh.cn
8th author	Daxin	Zhou		zhou.daxin@zs-hospital.sh.cn
9th author	Dehong	Kong (CA)		kong.dehong@zs-hospital.sh.cn
10th author	Jiarong	Zhang (CA)	Zhongshan Hospital Fudan University	zhang.jiarong@zs-hospital.sh.cn

Please use an additional sheet if there are more than 10 authors.

11th author	Qianzhou	Lv (CA)	Zhongshan Hospital Fudan University	lv.qianzhou@zs-hospital.sh.cn
12th author	Lihua	Guan (CA)		guanlihua2023@163.com
13th author	Junbo	Ge		jbge@zs-hospital.sh.cn

SPRINGER NATURE Springer Nature is one of the world's leading global research, educational and professional publishers, created in May 2015 through the combination of Nature Publishing Group, Palgrave Macmillan, Macmillan Education and Springer Science+Business Media.

SPRINGER NATURE Change of authorship request form - Journals (pre-acceptance)

Section 5: Author contribution, Acknowledgement and Disclosures. Please use this section to provide a new disclosure statement and, if appropriate, acknowledge any contributors who have been removed as authors and ensure you state what contribution any new authors made (if applicable per the journal or book (series) policy). Please ensure these are updated in your manuscript - after approval of the change(s) - as our production department will not transfer the information in this form to your manuscript.

New acknowledgements:

We would like to express our sincere gratitude to Professor D. K. for his valuable suggestions on the study design and manuscript framework. We are also thankful to the team of the Department of Echocardiography for their assistance in data collection, quality control, and measurement of key parameters.

New Disclosures (financial and non-financial interests, funding):

6.4 Conflict of interest

None declared.

6.5 Funding

This work was supported by National Key Research and Development Program of China (2022YFC2703902) and Natural Science Foundation of Shanghai (24ZR1410500).

New Author Contributions statement (if applicable per the journal policy):

L. G., Q. L., J. Z., and D. K. conceived, designed, and supervised the entire study. D. T., F. Y., and Y. Z. collected the data. D. T., D. L., Q. J., and D. K. performed the data analyses and data visualization. D. T., D. C., and M. T. performed the main experiments and wrote the manuscript. D. Z., Q. L., and J. G. provided assistance and revised the manuscript. L. G. acquired the funding. All authors provided critical comments and approved the final manuscript.

State 'Not applicable' if there are no new authors.

SPRINGER NATURE Springer Nature is one of the world's leading global research, educational and professional publishers, created in May 2015 through the combination of Nature Publishing Group, Palgrave Macmillan, Macmillan Education and Springer Science+Business Media.

SPRINGER NATURE

Change of authorship request form - Journals (pre-acceptance)

Section 6: Declaration of agreement. *All* authors, unchanged, new and removed *must* sign this declaration.
(NB: Please print the form, (docu)-sign and return/upload a scanned copy. Please note that signatures that have been inserted as an image file are acceptable as long as it is handwritten. Typed names in the signature box are unacceptable.) *** Please delete as appropriate. Delete all of the bold if you were on the original authorship list and are remaining as an author.**

	First name	Family name		Signature	Date
1st author	Dan	Tian	I agree to the proposed new authorship shown in section 4 **/and the addition/removal*of my name to the authorship list** /and the proposed change in corresponding author	Dan Tian	18-Dec-2025
2nd author	Dandan	Chen	I agree to the proposed new authorship shown in section 4 **/and the addition/removal*of my name to the authorship list** /and the proposed change in corresponding author	Dandan Chen	19-Dec-2025.
3rd author	Min	Tang	I agree to the proposed new authorship shown in section 4 **/and the addition/removal*of my name to the authorship list** /and the proposed change in corresponding author	Min Tang	19-Dec-2025
4th authors	Dawei	Lin	I agree to the proposed new authorship shown in section 4 **/and the addition/removal*of my name to the authorship list** /and the proposed change in corresponding author	Dawei Lin	19-Dec-2025
5th author	Qi	Jin	I agree to the proposed new authorship shown in section 4 **/and the addition/removal*of my name to the authorship list** /and the proposed change in corresponding author	Qi Jin	19-Dec-2025
6th author	~~Yang~~ Fan	Yang	I agree to the proposed new authorship shown in section 4 **/and the addition/removal*of my name to the authorship list** /and the proposed change in corresponding author	Fan Yang	19-Dec-2025
7th author	Yang	Zhan	I agree to the proposed new authorship shown in section 4 **/and the addition/removal*of my name to the authorship list** /and the proposed change in corresponding author	Yang Zhan	19-Dec-2025

SPRINGER NATURE Springer Nature is one of the world's leading global research, educational and professional publishers, created in May 2015 through the combination of Nature Publishing Group, Palgrave Macmillan, Macmillan Education and Springer Science+Business Media.

SPRINGER NATURE

Change of authorship request form - Journals (pre-acceptance)

Section 6: Declaration of agreement. *All* authors, unchanged, new and removed *must* sign this declaration.
(NB: Please print the form, (docu)-sign and return/upload a scanned copy. Please note that signatures that have been inserted as an image file are acceptable as long as it is handwritten. Typed names in the signature box are unacceptable.) *** Please delete as appropriate. Delete all of the bold if you were on the original authorship list and are remaining as an author.**

	First name	Family name		Signature	Date
8th author	Daxin	Zhou	I agree to the proposed new authorship shown in section 4 **/and the addition/removal*of my name to the authorship list** /and the proposed change in corresponding author	Daxin Zhou	19-Dec-2025
9th author	De hong	Kong	I agree to the proposed new authorship shown in section 4 **/and the addition/removal*of my name to the authorship list** /and the proposed change in corresponding author	De hong Kong	19-Dec-2025
10th author	Jiarong	Zhang	I agree to the proposed new authorship shown in section 4 **/and the addition/removal*of my name to the authorship list** /and the proposed change in corresponding author	Jiarong Zhang	19-Dec-2025
11th authors	Qianzhou	Lv	I agree to the proposed new authorship shown in section 4 **/and the addition/removal*of my name to the authorship list** /and the proposed change in corresponding author	Qianzhou Lv	19-Dec-2025
12th author	Lihua	Guan	I agree to the proposed new authorship shown in section 4 **/and the addition/removal*of my name to the authorship list** /and the proposed change in corresponding author	Lihua Guan	19-Dec-2025
13th author	Junbo	Ge	I agree to the proposed new authorship shown in section 4 **/and the addition/removal*of my name to the authorship list** /and the proposed change in corresponding author	Junbo Ge	08-Dec-2025
14th author			I agree to the proposed new authorship shown in section 4 **/and the addition/removal*of my name to the authorship list** /and the proposed change in corresponding author		

SPRINGER NATURE Springer Nature is one of the world's leading global research, educational and professional publishers, created in May 2015 through the combination of Nature Publishing Group, Palgrave Macmillan, Macmillan Education and Springer Science+Business Media.

SPRINGER NATURE **Change of authorship request form - Journals** (pre-acceptance)

	First name	Family name		Signature	Date
15th author			I agree to the proposed new authorship shown in section 4 **/and the addition/removal*of my name to the authorship list** /and the proposed change in corresponding author		
16th author			I agree to the proposed new authorship shown in section 4 **/and the addition/removal*of my name to the authorship list** /and the proposed change in corresponding author		
17th author			I agree to the proposed new authorship shown in section 4 **/and the addition/removal*of my name to the authorship list** /and the proposed change in corresponding author		

Please use an additional sheet if there are more than 10 authors.

In case of author collaborations with formal agreement:

	Name of consortium/consortia	First name	Family name		Signature	Date
Representative/legal guarantor		Lihua	Guan	I agree to the proposed new authorship shown in section 4 **/and the addition/removal*of my name to the authorship list** /and the proposed change in corresponding author	Lihua Guan	19-Dec-2015

Both added/removed authors should complete the information in the first table under Section 6.

---- End of form ----

SPRINGER NATURE Springer Nature is one of the world's leading global research, educational and professional publishers, created in May 2015 through the combination of Nature Publishing Group, Palgrave Macmillan, Macmillan Education and Springer Science+Business Media.
